# Observing a scale anomaly and a universal quantum phase transition in graphene

**DOI:** 10.1038/s41467-017-00591-8

**Published:** 2017-09-11

**Authors:** O. Ovdat, Jinhai Mao, Yuhang Jiang, E. Y. Andrei, E. Akkermans

**Affiliations:** 1 Department of Physics, Technion, Israel Institute of Technology, Haifa, 3200003 Israel; 2 Department of Physics and Astronomy, Rutgers University, Piscataway, NJ 08854 USA

## Abstract

One of the most interesting predictions resulting from quantum physics, is the violation of classical symmetries, collectively referred to as anomalies. A remarkable class of anomalies occurs when the continuous scale symmetry of a scale-free quantum system is broken into a discrete scale symmetry for a critical value of a control parameter. This is an example of a (zero temperature) quantum phase transition. Such an anomaly takes place for the quantum inverse square potential known to describe ‘Efimov physics’. Broken continuous scale symmetry into discrete scale symmetry also appears for a charged and massless Dirac fermion in an attractive 1/*r* Coulomb potential. The purpose of this article is to demonstrate the universality of this quantum phase transition and to present convincing experimental evidence of its existence for a charged and massless fermion in an attractive Coulomb potential as realized in graphene.

## Abstract (web summary)

When the continuous scale symmetry of a quantum system is broken, anomalies occur which may lead to quantum phase transitions. Here, the authors provide evidence for such a quantum phase transition in the attractive Coulomb potential of vacancies in graphene, and further envision its universality for diverse physical systems.

## Introduction

**Fig. 1:**
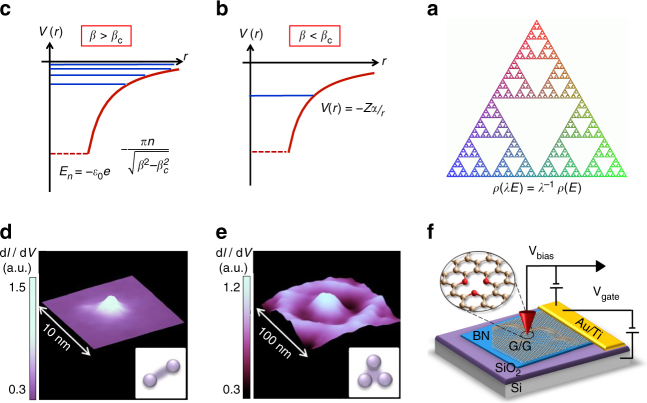
Schematic visualization of the purpose and main results of this paper. **a** Sierpinski gasket as typical featuring of such iterative fractal structures. This QPT is realized experimentally by creating single-atom vacancies in graphene. The function *ρ*(*E*) is the density of states and obeys a scaling relation characterising the existence of discrete scale symmetry. **b**, **c** Illustration of the universal quantum phase transition (QPT) obtained by varying the dimensionless parameter *β* ≡ *Zα* (see text for precise definitions) in the low-energy spectrum of a massless fermion in a Coulomb potential *V* = −*Zα*/*r* created by a charge *Z*. **b** For low values, *β* < *β*_c_, there is a single quasi-bound state close to zero energy. **c** For overcritical values, *β* > *β*_c_, the low-energy spectrum is a ladder *E*_*n*_ characterized by a discrete scale symmetry {*E*_*n*_} = {*λE*_*n*_} for $\lambda = {\rm{exp}}( {\pi {\rm{/}}\sqrt {{\beta ^{\rm{2}}} - \beta _{\rm{c}}^{\rm{2}}} } )$. **d**, **e** Experimental d*I*/d*V* maps of charged vacancy for fixed *β* < *β*_c_ (**d**) and *β* > *β*_c_ (**e**). The images illustrate the characteristic probability density of the resonances in (**b**, **c**). **f** Scanning tunnelling microscopy (*STM*) setup. Local charge *Z* is accumulated at the single vacancy in graphene by applying voltage pulses to the STM tip

Continuous scale symmetry (CS)—a common property of physical systems—expresses the invariance of a physical quantity *f*(*x*) (e.g., the mass) when changing a control parameter *x* (e.g., the length). This property is expressed by a simple scaling relation, *f*(*ax*) = *bf*(*x*), satisfied ∀*a* > 0 and corresponding *b*(*a*), whose general solution is the power law *f*(*x*) = *Cx* ^*α*^ with *α* = ln *b*/ln *a*. Other physical systems possess the weaker discrete scale symmetry (DS) expressed by the same aforementioned scaling relation but now satisfied for fixed values (*a*, *b*) and whose solution becomes *f*(*x*) = *x* ^*α*^ *G*(ln *x*/ln *a*), where *G*(*u* + 1) = *G*(*u*) is a periodic function. Physical systems having a DS are also known as self-similar fractals^1^ (Fig. 1a). It is possible to break CS into DS at the quantum level, a result which constitutes the basis of a special kind of scale anomaly^2, 3^.

A well-studied example is provided by the problem of a particle of mass *μ* in an attractive inverse square potential^4, 5^, which plays a role in various systems^6–9^ and more importantly in Efimov physics^10, 11^. Although well defined classically, the quantum mechanics of the scale—and conformal^12^—invariant Hamiltonian *H* = −Δ/2*μ* − *ξ*/*r* ^2^ (with *ħ* = 1) is well posed, but for large enough values of *ξ*, *H* is no longer self-adjoint^13, 14^. The corresponding Schrödinger equation for a normalisable wave function *ψ*(*r*) of energy *k* ^2^ = −2*μE* is,

1 $$\psi ''(r) + \frac{{d - 1}}{r}\psi '(r) + \frac{\zeta }{{{r^2}}}\psi (r) = {k^2}\psi (r),$$

where *ζ* ≡ 2*μξ* − *l*(*l* + *d* − 2) is a dimensionless parameter, *d* the space dimensionality and *l* the orbital angular momentum. Equation (1) is invariant under the transformation *r* → *λr* and *k* → *k*/*λ*, ∀*λ* (CS), namely to every normalisable wave function of energy *k* ^2^ corresponds a continuous family of states with energies (*λk*)^2^, so that the bound spectrum is a continuum unbounded from below. Various ways exist to cure this problem, based on cutoff regularisation and renormalisation group^15–21^, and all lead for the low-energy spectrum to a quantum phase transition (QPT) monitored by *ζ*, between a single bound state for *ζ* < *ζ*_c_ to an infinite and discrete energy spectrum for *ζ* > *ζ*_c_, independent of the regularisation procedure and given by

2 $${k_n}(\zeta ) = {\epsilon _0}{\kern 1pt} {e^{ - \frac{{\pi n}}{{\sqrt {\zeta - {\zeta _{\rm{c}}}} }}}},\,n \in {\Bbb Z},$$

which clearly displays DS. The critical value *ζ*_c_ = (*d* − 2)^2^/4 depends on the space dimensionality only, and ${\epsilon _{\rm{0}}}$ is a regularization dependent energy scale. In the overcritical phase *ζ* > *ζ*_c_, the corresponding renormalization group solution provides a rare example of a limit cycle^15, 16, 22^. Building on the previous example, it can be anticipated that the problem of a massless Dirac fermion in an attractive Coulomb potential^23–25^, −*Zα*/*r*, is also scale invariant (CS) and that the spectrum of resonant quasi-bound states presents similar features and a corresponding QPT.

In this work, we demonstrate the existence of such a universal QPT for arbitrary space dimension *d* ≥ 2 and independently of the short distance regularisation. We obtain an explicit formula for the low-energy fractal spectrum in the overcritical regime. In contrast to the Schrödinger case equation (1), the massless Dirac Hamiltonian displays an additional parity symmetry which may be broken by the regularisation. In that case, the degeneracy of the overcritical fractal spectrum is removed and two intertwined geometric ladders of quasi-bound states appear in the *s*-wave channel. All these features are experimentally demonstrated using a charged vacancy in graphene. We observe the overcritical spectrum and we obtain an experimental value for the universal geometric ladder factor in full agreement with the theoretical prediction. We also explain the observation of two intertwined ladders of quasi-bound states as resulting from the breaking of parity symmetry. Finally, we relate our findings to Efimov physics as measured in cold atomic gases.

## Results

### The Dirac model

The Dirac equation of a massless fermion in the presence of a −*Zα*/*r* potential is obtained from the Hamiltonian (with *ħ* = *c* = 1),

3 $$H = - i{\gamma ^0}{\gamma ^j}{\partial _j} - \frac{\beta }{r},$$

where (*γ* ^0^, *γ* ^*j*^) are Dirac matrices. Here the dimensionless parameter monitoring the transition is *β* = *Zα*, where *Z* is the Coulomb charge and *α* the fine structure constant. The QPT occurs at the critical value *β*_c_ = (*d* − 1)/2 (S﻿up﻿plementary Note 1) (A related anomalous behavior in the Dirac Coulomb problem has been identified long ago^26^ but its physical relevance was marginal since it required non existent heavy-nuclei Coulomb charges *Z* ≃ 1/*α* ≃ 137 to be observed. Moreover, the problem of a massive Dirac particle is different due to the existence of a finite gap which breaks CS.). For resonant quasi-bound states, we look for scattering solutions of the form *ψ*_in_ + *e* ^2*iη*^ *ψ*_sc_, where *η*(*E*) is the energy-dependent scattering phase shift and *ψ*_in,sc_(*r*, *E*) are two component objects representing the radial part of the Dirac spinor which behave asymptotically as,

4 $${\psi _{{\rm{in,sc}}}}(r,E) = {r^{\frac{{1 - d}}{2}}}\left( {{V_{{\rm{in,sc}}}}{\kern 1pt} {\kern 1pt} {{(2i{\rm{|}}E{\rm{|}}r)}^{ \mp i\beta }}{\kern 1pt} {e^{ \mp iEr}}} \right)$$

for $\left| E \right|r \gg 1$ and, using $\gamma \equiv \sqrt {{\beta ^2} - \beta _{\rm{c}}^2}$,

5 $${\psi _{{\rm{in,sc}}}}(r,E) = {r^{\frac{{1 - d}}{2}}}\left( {U_{{\rm{in,sc}}}^ - {\kern 1pt} {{(2iEr)}^{ - i\gamma }} + U_{{\rm{in,sc}}}^ + {{(2iEr)}^{i\gamma }}} \right),$$

for $\left| E \right|r \ll 1$ and for the lowest angular momentum channels. The two component objects *V*_in,sc_ and $U_{{\rm{in,sc}}}^ \pm$ in Eqs. (4) and (5) are constants. It is easy to infer from (5) that *β* *=* *β*_c_ plays a special role. Indeed for *β* *>* *β*_c_, there exists a family of normalisable solutions that admit complex eigenvalues *E* = −*i* $\epsilon$, hence the Hamiltonian (3) is not self-adjoint (*H* ≠ *H* ^†^). To properly define this quantum problem, a regularisation is thus needed for the too strong potential at overcritical values of *β* = *Zα*. This is achieved by introducing a cutoff length *L* and a boundary condition at *r* = *L*, which is equivalent to replacing the Coulomb potential at short distances by a well-behaved potential whose exact form is irrelevant in the low-energy regime $EL \ll 1$. The resulting mixed boundary condition can be written as $h = {\left. {{\Psi _2}\left( {r,E} \right){\rm{/}}{\Psi _1}\left( {r,E} \right)} \right|_{r \to {L^ + }}}$, where Ψ_1,2_ represent the two components of the aforementioned radial part of the Dirac spinor. The resulting scattering phase shift *η*(*E*, *L*, *h*), which contains all the information about the regularisation, thus becomes a function of *L* and of the parameter *h*. The quasi-bound states energy spectrum is obtained from the scattering phase shift by means of the Krein–Schwinger relation^27, 28^ which relates the change of density of states *δρ* to the energy derivative of *η*, (This is also related to the Wigner time delay^29^ and to the Friedel sum rule)

6 $$\delta \rho (E) = \frac{1}{\pi }\frac{{{\rm{d}}\eta (E)}}{{{\rm{d}}E}}{\kern 1pt} .$$

### Theoretical structure of quasi-bound spectrum

**Fig. 2:**
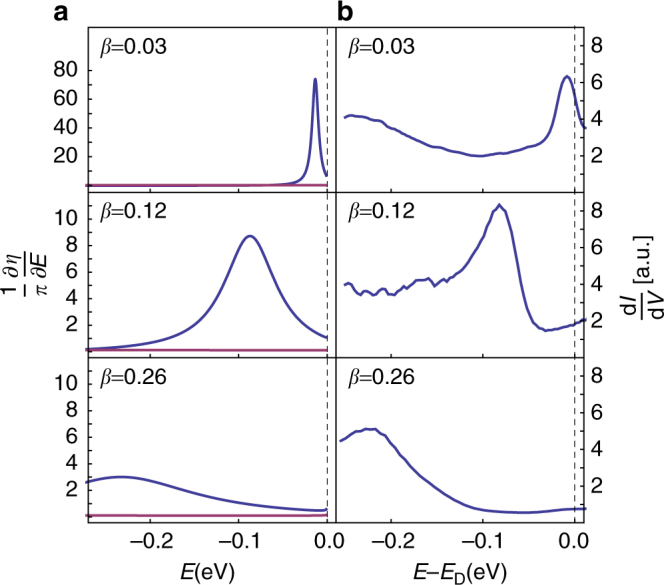
Experimental and theoretical picture in the undercritical regime. **a** Theoretical behavior of (1)/(*π*)d*η*/d*E* for *d* = 2 showing quasi-bound states of a massless Dirac fermion in the undercritical regime *β* < 1/2. In the scale-free low-energy, $EL \ll 1$ regime, the *m* = −1 (*blue*) branch contains a single peak and the *m* = 0 (purple) branch shows no peak independently of the choice of boundary condition (see Supplementary Note 2). While increasing *β*, the resonance shifts to lower energy and becomes broader. **b** Excitation spectrum measured in graphene using STM as a function of the applied voltage *V*. The determination of the parameter *β* is explained in the text

**Fig. 3:**
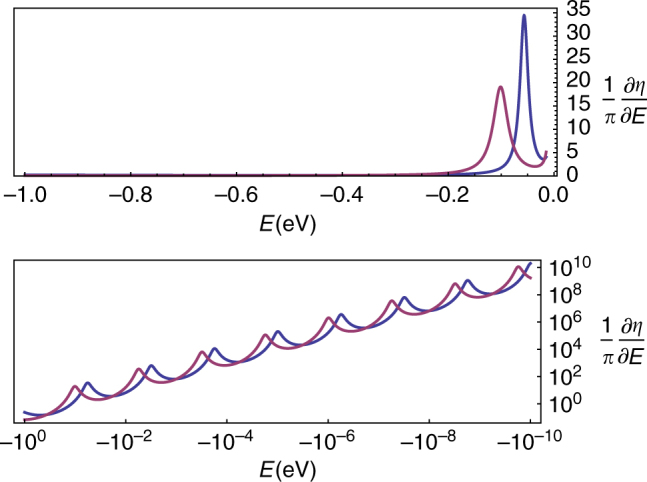
Theoretical behavior of the low energy and scale-free part of the quasi-bound states spectrum in the overcritical regime for *d* = 2 and *β* = 1.2 > *β*_c_( = 1/2). The lower plot displays the detailed structure of the infinite geometric ladders. Note that the *m* = −1 (*blue*) and *m* = 0 (*purple*) ladders are intertwined. These results are independent of the boundary condition

From now on, and to compare to experimental results further discussed, we consider the case *d* = 2, for which there is a single orbital angular momentum quantum number $m \in {\Bbb Z}$. The corresponding critical coupling becomes *β*_c_ = |*m* + 1/2| ≥ 1/2, giving rise to the *s*-wave channels, *m* = 0, −1, for which *β*_c_ = 1/2. Depending on the choice of boundary condition *h*, *δρ*(*E*) can be degenerate or non-degenerate over these two *s*-wave channels. This degeneracy originates from the symmetry of the (2 + 1) Dirac Hamiltonian (3) under parity, (*x*, *y*) → (−*x*, *y*), and its existence is equivalent to whether or not the boundary condition breaks parity (Supplementary Note 2). In what follows, we will consider the generic case in which there is no degeneracy. In the undercritical, *β* < *β*_c_, and low-energy regime $EL \ll 1$, we observe (Figs. 1b, 2a) a single quasi-bound state originating from only one of the *s*-wave channels and which broadens as *β* increases. In the overcritical regime *β* > *β*_c_, this picture changes dramatically. (We emphasize that this picture remains valid for all values of *β* > *β*_c_ and not only in the vicinity of *β*_c_). The low-energy ($EL \ll 1$) scattering phase shift displays two intertwined, infinite geometric ladders of quasi-bound states (Figs. 1c, 3) at energies *E*_*n*_ still given by (2) but with *ζ* − *ζ*_c_ now replaced by ${\beta ^2} - \beta _{\rm{c}}^2$. (Moreover, note that the energy scale ${\epsilon _0}$ for the Dirac case is different from the inverse square Schrödinger case defined in equation (1)). This sharp transition at *β*_c_ belongs to the same universality class as presented for the inverse square Schrödinger problem, namely CS of the quasi-bound states spectrum is broken for *β* > *β*_c_ into a DS phase characterized by a fractal distribution of quasi-bound states. The QPT thus reflects the lack of self-adjointness of the Hamiltonian equation (3) and the necessary regularisation procedure leads to a scale anomaly in which CS is broken into DS.

### Experimental realization in graphene

A particularly interesting condensed matter system, where the previous considerations seem to be relevant is graphene in the presence of implanted Coulomb charges in conveniently created vacancies^30^. It is indeed known that low-energy excitations in graphene behave as a massless Dirac fermion field with a linear dispersion $\epsilon$ = ±*v*_F_|*p*| and a Fermi velocity *v*_F_ ≃ 10^6^ m/s^31^. These characteristics have been extensively exploited to make graphene a very useful platform to emulate specific features of quantum field theory, topology and especially QED^23^, since an effective fine structure constant *α*_*G*_ ≡ *e* ^2^/*ħv*_F_ of order unity is obtained by replacing the velocity of light *c* by *v*_F_.

It has been recently shown that single-atom vacancies in graphene can stably host local charge^30^. Density functional theory calculations have shown that, when a carbon atom is removed from the honeycomb lattice, the atoms around the vacancy site rearrange into a lower energy configuration^32^. The resulting lattice reconstruction causes a charge redistribution, which in the ground state has an effective local charge of ≈+1. Recent Kelvin probe force microscopy measurements of the local charge at the vacancy sites are in good agreement with the Density functional theory predictions. Vacancies are generated by sputtering graphene with He^+^ ions^33, 34^. Charge is modified and measured at the vacancy site by means of scanning tunnelling spectroscopy and Landau level spectroscopy as detailed in ref. ^30^. Applying multiple pulses allows for a gradual increase in the vacancy charge, which in turn acts as an effective tunable Coulomb source. Moreover, the size of the source inside the vacancy is small (≈1 nm) as compared to the method of deposited metal clusters^35^. Using this method, we are able to observe the transition expected to occur at *β* = 1/2 and to measure and analyze three resonances for a broad range of *β* values.

To establish a relation between the measured differential conductance and the spectrum of quasi-bound states, we recall that the tunnel current *I*(*V*) is proportional to both the density of states *ρ*_*t*_($\epsilon$) of the STM tip and *ρ*($\epsilon$) of massless electronic excitations in graphene at the vacancy location. We also assume that the tunnel matrix element |*t*|^2^ depends only weakly on energy and that both voltage and temperature are small compared to the Fermi energy and height of the tunnelling potential, so that the current *I*(*V*) = *G*_t_ *V* is linear with *V* thus defining the tunnel conductance *G*_t_ = 2*π*(*e* ^2^/*ħ*)|*t*|^2^ *ρ*_*t*_ *ρ*($\epsilon$). Assuming that *ρ*_*t*_ of the reference electrode (the tip) is energy independent, a variation *δρ*($\epsilon$) of the local density of states at the vacancy leads to a variation *δI*(*V*) of the current and thus to a variation *δG*_t_(*V*) of the tunnel conductance so that, at zero temperature, we obtain^36^

7 $$\frac{{\delta {G_{\rm{t}}}(V)}}{{{G_{\rm{t}}}}} = \frac{{\delta \rho (\epsilon )}}{{{\rho _0}}}{\kern 1pt} ,$$

where *ρ*_0_ is the density of states in the absence of vacancy. By considering the vacancy as a local perturbation, each quasi-particle state is characterized by its scattering phase shift taken to be the phase shift *η*(*E*) of the quasi-bound Dirac states previously calculated. Then, the change of density of states *δρ*(*E*) is obtained from equation (6) and combining together with equation (7) leads to the relation,

8 $$\frac{{{\rm{d}}\delta I}}{{{\rm{d}}V}} = \frac{{{G_{\rm{t}}}}}{{\pi {\rho _0}}}\frac{{{\rm{d}}\eta (E)}}{{{\rm{d}}E}}$$

between the differential tunnel conductance and the scattering phase shift.

**Fig. 4:**
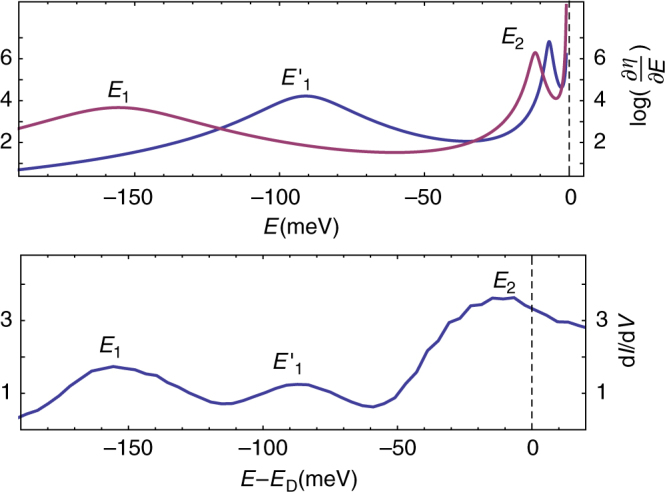
Experimental and theoretical picture in the overcritical regime. *Upper plot*: Theoretical behavior of the low energy and scale-free part of the overcritical (*β* = 1.33) quasi-bound states spectrum obtained from (6). The *blue* (*purple*) *line* corresponds to *m* = −1 (*m* = 0). *Lower plot*: Experimental values of the (STM) tunnelling conductance measured at the position of charged vacancies in graphene. The labelling *E*_1_, *E*_2_, $E_1^\prime$ of the peaks is explained in the text

**Fig. 5:**
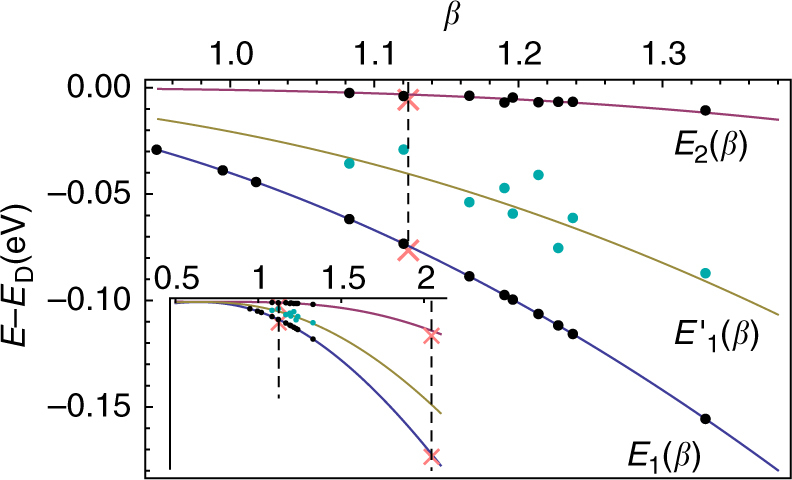
Behavior of the energies *E*_*n*_(*β*) of the quasi-bound state spectrum. The curves are obtained from (2) for *E*_1_(*β*), $E_1^\prime$(*β*), *E*_2_(*β*) as adapted to the massless Dirac case. The *black* and *cyan dots* correspond to the values measured in graphene. The two *pink x*’s are the values of Efimov energies measured in Caesium atoms^39, 47^, which corresponds to the (overcritical) fixed Efimov value *β*_E_ = 1.1236. Additional experimental points obtained in refs ^40, 41^ are displayed in the *inset*

**Fig. 6:**
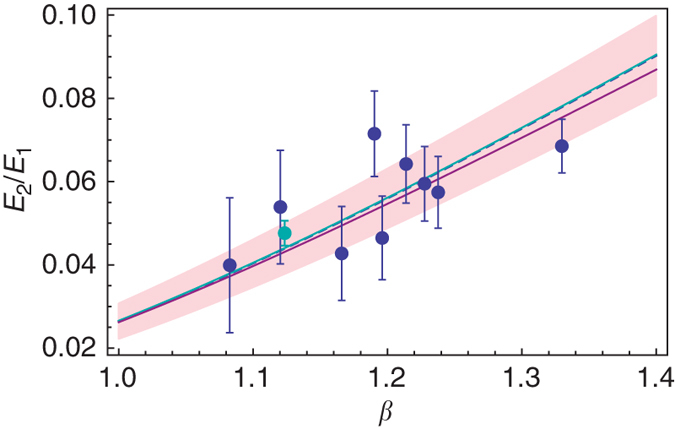
Comparison between the experimentally obtained *E*_2_/*E*_1_ ratio and the universal factor ${e^{ - \pi /\sqrt {{\beta ^2} - 1/4} }}$. *Blue points*: the ratio *E*_2_/*E*_1_ obtained from the position of the points in Fig. 5. Cyan point: Universal Efimov energy ratio as measured in Caesium atoms^39, 47^. *Blue line* (*dashed*): the corresponding optimized curve, fitted according to the model ${e^{ - b/\sqrt {{\beta ^2} - 1/4} }}$ and corresponding to *b* = 3.145 with standard error of Δ*b* = 0.06 consistent with the predicted value *π*. The *shaded pink* region is the ±2Δ*b* confidence interval of the curve. *Cyan* line: universal low-energy factor ${e^{ - \pi /\sqrt {{\beta ^2} - 1/4} }}$. *Purple line*: theoretical ratio *E*_2_/*E*_1_ obtained from the exact solution of the Dirac equation. As *β* → 0.5, |*E*_*n*_| becomes smaller therefore the green and purple curves coincide for low *β*. The *error bar* on the resonance energies is ±1 mV

The measurements and the data analysis presented here were carried out as follows: positive charges are gradually injected into an initially prepared single atom vacancy and the differential conductance *δG*_t_(*V*) is measured at each step as a function of voltage. Since we are looking at the positions of resonant quasi-bound states, both quantities displayed in Figs. 2, 4 give the same set of resonant energies, independently of the energy-independent factor *G*_t_/*πρ*_0_. For low enough values of the charge, the differential conductance displayed in Fig. 2b, shows the existence of a single quasi-bound state resonance. The behavior close to the Dirac point, namely in the low-energy regime independent on the short distance regularization, is very similar to the theoretical prediction of Fig. 2a. When the build up charge exceeds a certain value, we note the appearance of three resonances, emerging out of the Dirac point. We interpret these resonances as the lowest overcritical (*β* > 1/2) resonances, which we denote *E*_1_, $E_1^\prime$, *E*_2,_ respectively. The corresponding theoretical and experimental behaviors displayed in Figs. 3, 4, show a very good qualitative agreement. To achieve a quantitative comparison solely based on the previous Dirac Hamiltonian equation (3), we fix *L* and the boundary condition *h* and deduce the theoretical *β* values corresponding to the respective positions of the lowest overcritical resonance *E*_1_ (as demonstrated in Fig. 4). This allows to determine the lowest branch *E*_1_(*β*) for *n* = 1 represented in Fig. 5. Then, the experimental points $E_1^\prime$, *E*_2_ are directly compared to their corresponding theoretical branch as seen in Fig. 5. We determine *L* and *h*, according to the ansatz *h* = *a*(*m* + 1), and obtain the best correspondence for *L* ≃ 0.2 nm, *a* ≃ −0.85. We compare the experimental *E*_2_/*E*_1_ ratio with the universal prediction ${E_{n + 1}}{\rm{/}}{E_n} = {e^{ - \pi /\sqrt {{\beta ^2} - 1/4} }}$ as seen in Fig. 6. A trend-line of the form ${e^{ - b/\sqrt {{\beta ^2} - 1/4} }}$ is fitted to the ratios *E*_2_/*E*_1_, yielding a statistical value of *b* = 3.145 with standard error of Δ*b* = 0.06 consistent with the predicted value *π*. An error of ±1 *mV* is assumed for the position of the energy resonances.

A few comments are appropriate: (i) The points on the *E*_2_(*β*) curve follow very closely the theoretical prediction ${E_{n + 1}}{\rm{/}}{E_n} = {e^{ - \pi /\sqrt {{\beta ^2} - 1/4} }}$. This result is insensitive to the choice of *h*, thus manifesting the universality of the ratio *E*_*n*+1_/*E*_*n*_. (ii) In contrast, the correspondence between the $E_1^\prime$ points and the theoretical branch is sensitive to the choice of *h*. This reflects the fact that while each geometric ladder is of the form equation (2) (with the appropriate *ζ* → *β* change), the energy scale ${\epsilon _{\rm{0}}}$ is different between the two thus leading to a shifted relative position of the two geometric ladders in Fig. 3. The ansatz taken for *h* is phenomenological (Supplementary Note 2), however, we find that in order to get reasonable correspondence to theory, the explicit dependence on *m* is needed. More importantly, it is necessary to use a degeneracy breaking boundary condition to describe the $E_1^\prime$(*β*) points. For instance, if the Coulomb potential is regularized by a constant potential for *r* ≤ *L* ^37^, then both angular momentum channels (i.e., the $E_1^\prime$ and *E*_1_ points) become degenerate. The existence of the experimental $E_1^\prime$ branch is therefore a distinct signal that parity symmetry in the corresponding Dirac description equation (3) is broken. In graphene, exchanging the triangular sublattices is equivalent to a parity transformation. Creating a vacancy breaks the symmetry between the two sub-lattices and is therefore at the origin of broken parity in the Dirac model. (iii) The value *L* ≃ 0.2 nm is fully consistent with the low-energy requirement ${E_1}L{\rm{/}}\hbar {v_{\rm{F}}} \simeq 0.03 \ll 1$ necessary to be in the regime relevant to observe the *β*-driven QPT.

## Discussion

A further argument in support of the universality of this QPT is achieved by comparing the experimental results obtained in graphene with those deduced from a completely different physical problem. To that purpose, we dwell for a short while recalling the basics underlying Efimov physics^38^. Back to 1970, Efimov^10^ studied the quantum problem of three identical nucleons of mass *m* interacting through a short range (*r*_0_) potential. He pointed out that when the scattering length *a* of the two-body interaction becomes very large, $a \gg {r_0}$, there exists a scale-free regime for the low-energy spectrum, ${\hbar ^2}{\rm{/}}m{a^2} \ll E \ll {\hbar ^2}{\rm{/}}mr_0^2$, where the corresponding bound-states energies follow the geometric series $\left( {\sqrt { - {E_n}} = - {{\tilde \epsilon }_0}{e^{ - \pi n/{s_0}}}} \right),$ where ${s_0} \simeq 1.00624$ is a dimensionless number and ${\tilde \epsilon _0}$ a problem-dependent energy scale. Efimov deduced these results from an effective Schrödinger equation in *d* = 3 with the radial (*l* = 0) attractive potential $V(r) = - \left( {s_0^2 + 1{\rm{/}}4} \right){\rm{/}}{r^2}$. Using Eqs. (1) and (2) and the critical value *ζ*_c_ = (*d* − 2)^2^/4 = 1/4 for this Schrödinger problem, we deduce the *ζ* value for the Efimov effect to be $s_0^2 + 1{\rm{/}}4 \, >\, {\zeta _{\rm{c}}}$ corresponding to the overcritical regime of the QPT. The value of *β* matching to the Efimov geometric series factor ${e^{\pi /{s_0}}}$ is ${\beta _{\rm{E}}} = \sqrt {s_0^2 + 1{\rm{/}}4} = 1.1236$, referred to as the fixed Efimov value. Despite being initially controversial, Efimov physics has turned into an active field especially in atomic and molecular physics where the universal spectrum has been studied experimentally^39–46^ and theoretically^38^. The first two Efimov states *E*_*n*_ (*n* = 1, 2) have been recently determined using an ultracold gas of caesium atoms^47^. Although the Efimov spectrum always lies at a fixed and overcritical value of the coupling, unlike the case of graphene where *β* can be tuned, the universal character of the overcritical regime allows nevertheless for a direct comparison of these two extremely remote physical systems. To that purpose, we include the Efimov value *β*_E_ in the expression obtained for the massless Dirac fermion in a Coulomb potential and insert the corresponding data points obtained for cold atomic caesium in the graphene plot (Fig. 5) up to an appropriate scaling of ${\tilde \epsilon _0}$. The results are fully consistent thus showing in another way the universality presented.

There are other remote examples of systems displaying this universal QPT, e.g., flavoured QED3^48^, and the XY model (Kosterlitz-Thouless^8^ and roughening transitions^22^). Our results provide a useful and original probe of characteristic features of this universal QPT and motivate a more thorough study of this transition.

## Methods

Our sample is stacked two layers of graphene on top of a thin BN flake (see Fig. 1f). The standard dry transfer procedure is followed to get this heterostructure. A large twisted angle between the two layers graphene is selected in order to weaken the coupling. The free-standing like feature for the top layer graphene is checked by the Landau levels spectroscopy. To achieve the diluted single vacancies, the sample is exposed to the helium ion beam for short time (100 eV for 5 s) followed by the high temperature annealing. The experiment is performed at 4.2 K with a home-built STM. The d*I*/d*V* (*I* is the current, *V* is the bias) is recorded by the standard lock-in technique, with a small AC modulation 2 mV at 473.1 Hz added on the DC bias. To tune the effective charge on the vacancy, we apply the voltage pulse (−2 V, 100 ms) with the STM tip directly locating on top of the vacancy.

### Data availability

The data that support the findings of this study are available from the corresponding author upon request.

## Electronic supplementary material

Supplementary Information

Peer Review File

## References

[1] Akkermans, E. in *Fractal Geometry and Dynamical Systems in Pure and Applied Mathematics II: Fractals in Applied Mathematics* vol. 601, 1–21 (eds Carf, D., Lapidus, M. L., Pearse, E. P. J. & van Frankenhuijsen, M.) (American Mathematical Society (AMS), 2013).

[2] Adler SL (1969). Axial-vector vertex in spinor electrodynamics. Phys. Rev..

[3] Bell JS, Jackiw R (1969). A PCAC puzzle: *π*^0^ → *γγ* in the *σ*-model. Il Nuovo Cimento A.

[4] Case KM (1950). Singular potentials. Phys. Rev..

[5] Landau, L. D. *Quantum Mechanics: Non-Relativistic Theory* (Butterworth-Heinemann, 1991).

[6] Lévy-Leblond J-M (1967). Electron capture by polar molecules. Phys. Rev..

[7] Camblong HE, Epele LN, Fanchiotti H, Garca Canal CA (2001). Quantum anomaly in molecular physics. Phys. Rev. Lett..

[8] Kaplan DB, Lee J-W, Son DT, Stephanov MA (2009). Conformality lost. Phys. Rev. D.

[9] Nisoli C, Bishop AR (2014). Attractive inverse square potential, *U*(1) gauge, and winding transitions. Phys. Rev. Lett..

[10] Efimov V (1970). Energy levels arising from resonant two-body forces in a three-body system. Phys. Lett. B.

[11] Efimov V (1971). Weakly-bound states of three resonantly-interacting particles. Sov. J. Nucl. Phys.

[12] Jackiw, R. W. *Diverse Topics in Theoretical and Mathematical Physics* (World Scientific, 1995).

[13] Meetz K (1964). Singular potentials in nonrelativistic quantum mechanics. Il Nuovo Cimento.

[14] Gitman, D. M., Tyutin, I. & Voronov, B. L. *Self-adjoint Extensions in Quantum Mechanics: General Theory and Applications to Schrödinger and Dirac Equations with Singular Potentials* vol. 62 (Springer, 2012).

[15] Albeverio S, Høegh-Krohn R, Wu TT (1981). A class of exactly solvable three-body quantum mechanical problems and the universal low energy behavior. Phys. Lett. A.

[16] Beane SR (2001). Singular potentials and limit cycles. Phys. Rev. A.

[17] Mueller, E. J. & Ho, T.-L. Renormalization group limit cycles in quantum mechanical problems. Preprint at arXiv:cond-mat/0403283 (2004).

[18] Braaten E, Phillips D (2004). Renormalization-group limit cycle for the 1/*r*^2^ potential. Phys. Rev. A.

[19] Hammer H-W, Swingle BG (2006). On the limit cycle for the 1/*r*^2^ potential in momentum space. Ann. Phys..

[20] Moroz S, Schmidt R (2010). Nonrelativistic inverse square potential, scale anomaly, and complex extension. Ann. Phys..

[21] De Martino A, Klöpfer D, Matrasulov D, Egger R (2014). Electric-dipole-induced universality for Dirac fermions in graphene. Phys. Rev. Lett..

[22] Kolomeisky EB, Straley JP (1992). Universality classes for line-depinning transitions. Phys. Rev. B.

[23] Shytov AV, Katsnelson MI, Levitov LS (2007). Atomic collapse and quasi-rydberg states in graphene. Phys. Rev. Lett..

[24] Pereira VM, Nilsson J, Castro Neto AH (2007). Coulomb impurity problem in graphene. Phys. Rev. Lett..

[25] Nishida Y (2014). Vacuum polarization of graphene with a supercritical Coulomb impurity: low-energy universality and discrete scale invariance. Phys. Rev. B.

[26] Pomeranchuk I, Smorodinsky J (1945). On the energy levels of systems with *Z* > 137. J. Phys..

[27] Akkermans E (1997). Twisted boundary conditions and transport in disordered systems. J. Math. Phys..

[28] Akkermans, E., Dunne, G. & Levy, E. in *Optics of Aperiodic Structures: Fundamentals and Device Applications* (ed. Negro, L. D.) (Pan Stanford Publishing, 2013).

[29] Smith FT (1960). Lifetime matrix in collision theory. Phys. Rev..

[30] Mao J (2016). Realization of a tunable artificial atom at a supercritically charged vacancy in graphene. Nat. Phys..

[31] Andrei EY, Li G, Du X (2012). Electronic properties of graphene: a perspective from scanning tunneling microscopy and magnetotransport. Rep. Prog. Phys..

[32] Liu Y, Weinert M, Li L (2015). Determining charge state of graphene vacancy by noncontact atomic force microscopy and first-principles calculations. Nanotechnology.

[33] Lehtinen O (2010). Effects of ion bombardment on a two-dimensional target: atomistic simulations of graphene irradiation. Phys. Rev. B.

[34] Chen J-H, Li L, Cullen WG, Williams ED, Fuhrer MS (2011). Tunable Kondo effect in graphene with defects. Nat. Phys..

[35] Wang Y (2013). Observing atomic collapse resonances in artificial nuclei on graphene. Science.

[36] Akkermans, E. & Montambaux, G. In *Mesoscopic Physics of Electrons and Photons*, ch. 7 (Cambridge University Press, 2007).

[37] Pereira VM, Kotov VN, Castro Neto AH (2008). Supercritical Coulomb impurities in gapped graphene. Phys. Rev. B.

[38] Braaten E, Hammer H-W (2006). Universality in few-body systems with large scattering length. Phys. Rep..

[39] Kraemer T (2006). Evidence for Efimov quantum states in an ultracold gas of caesium atoms. Nature.

[40] Tung S-K, Jiménez-Garca K, Johansen J, Parker CV, Chin C (2014). Geometric scaling of Efimov states in a ^6^Li–^133^Cs mixture. Phys. Rev. Lett..

[41] Pires R (2014). Observation of Efimov resonances in a mixture with extreme mass imbalance. Phys. Rev. Lett..

[42] Pollack SE, Dries D, Hulet RG (2009). Universality in three- and four-body bound states of ultracold atoms. Science.

[43] Gross N, Shotan Z, Kokkelmans S, Khaykovich L (2009). Observation of universality in ultracold ^7^Li three-body recombination. Phys. Rev. Lett..

[44] Lompe T (2010). Radio-frequency association of Efimov trimers. Science.

[45] Nakajima S, Horikoshi M, Mukaiyama T, Naidon P, Ueda M (2011). Measurement of an Efimov trimer binding energy in a three-component mixture of ^6^Li. Phys. Rev. Lett..

[46] Kunitski M (2015). Observation of the Efimov state of the helium trimer. Science.

[47] Huang B, Sidorenkov LA, Grimm R, Hutson JM (2014). Observation of the second triatomic resonance in Efimov’s scenario. Phys. Rev. Lett..

[48] Appelquist T, Nash D, Wijewardhana LCR (1988). Critical behavior in (2 + 1)-dimensional QED. Phys. Rev. Lett..

