# Supplementary material for: Observing a scale anomaly and a universal quantum phase transition in graphene
Source: Nat Commun. 2017 Sep 11;8:507. doi: 10.1038/s41467-017-00591-8 (PMC5593936; doi:10.1038/s41467-017-00591-8)
Supplement: Supplementary file 1 — Supplementary Information [file 41467_2017_591_MOESM1_ESM.pdf]

Description of Supplementary Files

File Name: Supplementary Information
Description: Supplementary Figures, Supplementary Notes, Supplementary Methods and Supplementary References

File Name: Peer Review File

We present in supplementary figures 1 and 2 all overcritical and undercritical measurements, and their corresponding theoretical plots. In supplementary figure 3 we present the STM topography image of an isolated vacancy in graphene.

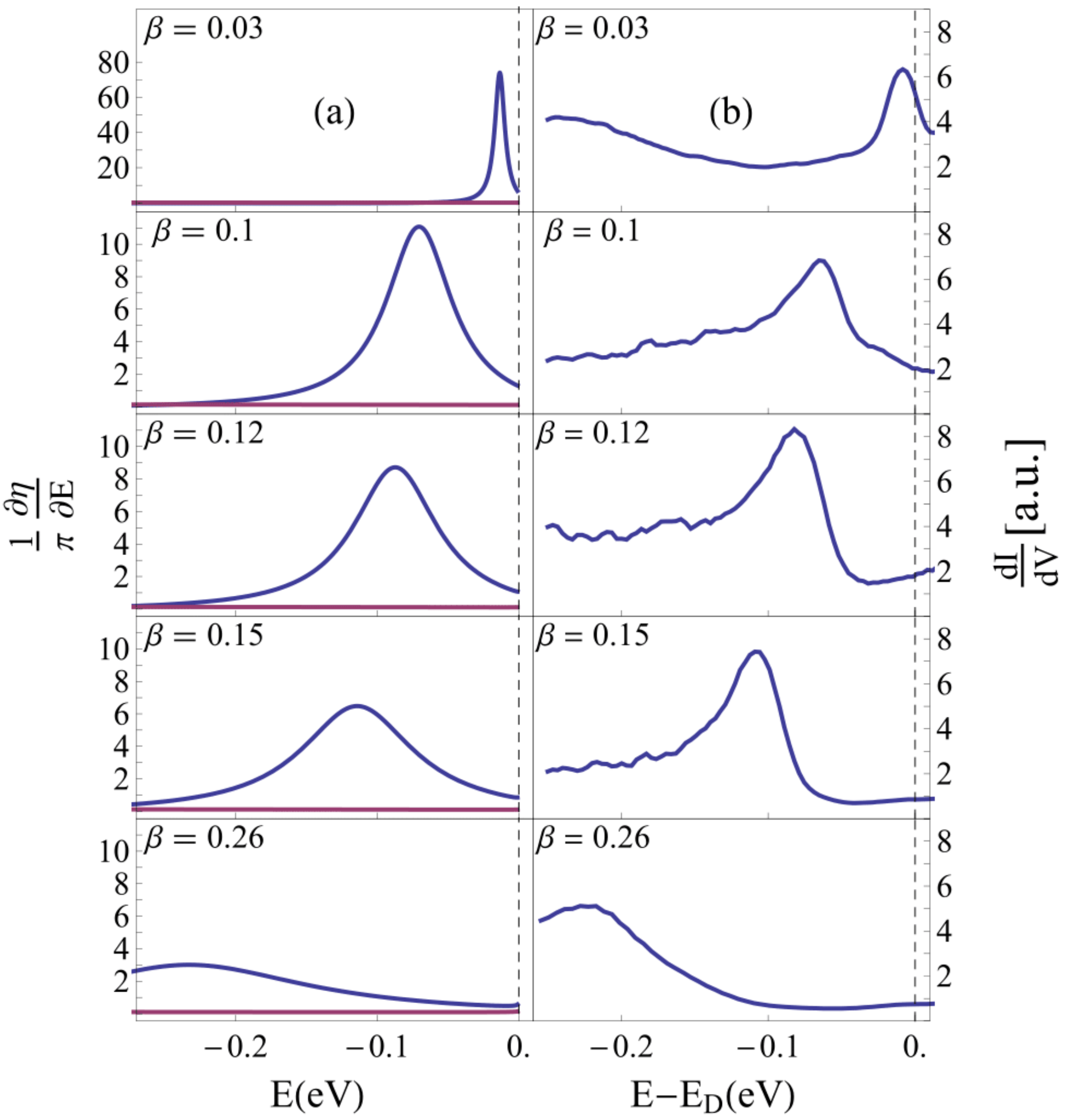

Supplementary Figure 1: **Experimental and theoretical picture in the undercritical regime.** a. Theoretical behaviour of the undercritical ($\beta < 1/2$) quasi-bound states spectrum of massless Dirac fermions. b. STM measurement of the quasi-particle spectrum at the position of the charged vacancy in the undercritical regime. See main text for more details.

Supplementary Note 1: Dirac Coulomb problem in d+1 dimensions – critical coupling, phase shift and quasi bound states

In what follows, we study a system described by a massless Dirac particle in the presence of an electric potential that has an inverse radial tail in $d+1$ dimensions. We show that beyond a critical coupling value, an anomalous breaking of conformal symmetry occurs and the system is described by a discrete scale invariant spectrum. We obtain for a general over critical coupling and short range behaviour of the potential, an expression for an infinite series of geometrically spaced quasi bound states.

The massless Dirac equation with an attractive potential $V(r) = -\frac{\beta}{r}$, $\beta \equiv Z\alpha$ in $d+1$ dimensions is

$$i\gamma^{\mu}(\partial_{\mu} + ieA_{\mu})\psi(x^{\nu}) = 0 \tag{1}$$

where $\mu = 0\ldots d$, A_{μ} is the electromagnetic potential (EM)

$$\begin{aligned} eA_0 &= -\beta/r \\ A_i &= 0 \quad i = 1,\ldots,d. \end{aligned} \tag{2}$$

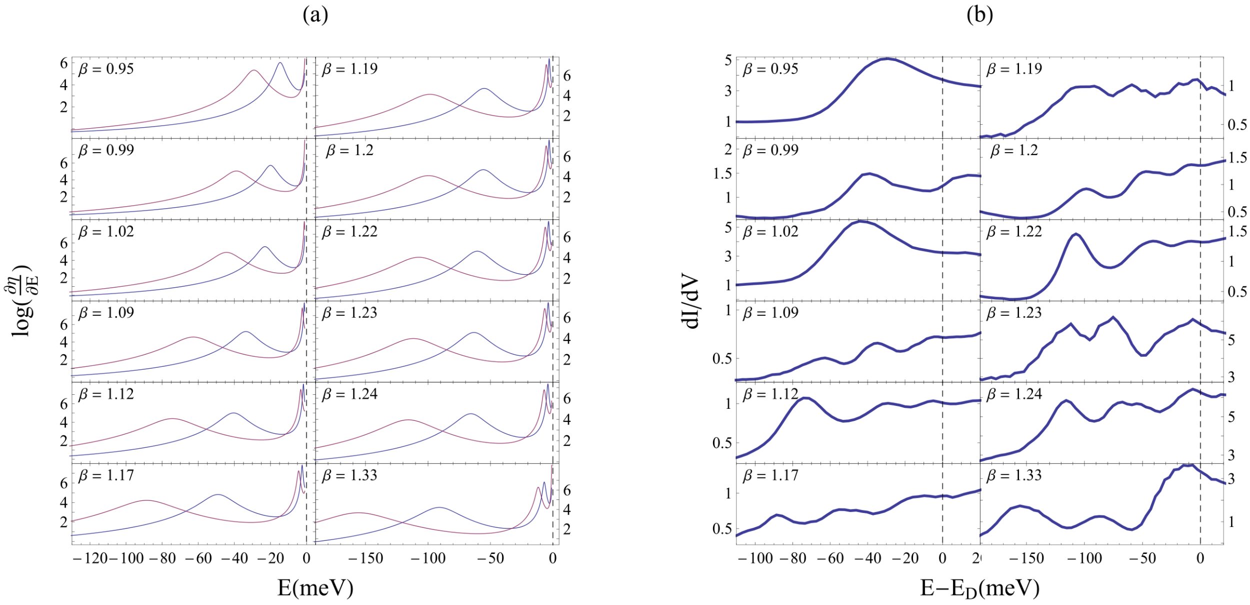

Supplementary Figure 2: **Experimental and theoretical picture in the overcritical regime.** a. Theoretical behaviour of the overcritical ($\beta > 1/2$) quasi-bound states spectrum of massless Dirac fermions. b. STM measurement of the quasiparticle spectrum at the position of the charged vacancy in the overcritical regime. See main text for more details.

and γ^μ are $d+1$ matrices satisfying the anti-commutation relation

$$\{\gamma^\mu, \gamma^\nu\} = 2\eta^{\mu\nu} \tag{3}$$

with $\eta_{\mu\nu}$ being the $d+1$ Minkowski metric with a 'mostly minus' sign convention. The Hamiltonian of the system is expressed as

$$H = \gamma^0\gamma^j p_j - \beta/r \tag{4}$$

where $j = 1 \dots d$ and corresponds to the scale invariant eigenvalue equation $H\psi = E\psi$ equivalent to (1).

Utilizing rotational symmetry, the angular part of supplementary equation (1) can be solved and the radial dependence of $\psi(x^\nu)$ is given in terms of two functions $\Psi_2(r), \Psi_1(r)$ [1] determined by the following set of equations

$$\begin{aligned} \Psi_2'(r) + \frac{(d-1+2K)}{2r}\Psi_2(r) &= \left(E + \frac{\beta}{r}\right)\Psi_1 \\ -\Psi_1'(r) - \frac{(d-1-2K)}{2r}\Psi_1(r) &= \left(E + \frac{\beta}{r}\right)\Psi_2 \end{aligned} \tag{5}$$

where

$$K \equiv \begin{cases} \pm\left(l + \frac{d-1}{2}\right) & d > 2 \\ m + 1/2 & d = 2 \end{cases}, \tag{6}$$

$l = 0, 1, \dots$ and $m \in \mathbb{Z}$ are orbital angular momentum quantum numbers. In terms of these radial functions, the scalar product of two eigenfunctions $\psi, \tilde{\psi}$ is given by

$$\int dV\, \psi^\dagger \tilde{\psi} = \int dr\, r^{d-1}\left(\Psi_1^*(r)\tilde{\Psi}_1(r) + \Psi_2^*(r)\tilde{\Psi}_2(r)\right).$$

We introduce a short distance radial cut-off L and assume that there exist an electric Coulomb potential $V(r) = -\frac{\beta}{r}$ for $r > L$ and some interaction at $r < L$ that can be modelled by a BC at $r = L$. The equivalent mixed boundary condition of (5) can be written as follows [2]

$$h = \left.\frac{\Psi_2(r)}{\Psi_1(r)}\right|_{r\to L^+} \tag{7}$$

where h is determined by the short range physics and in general can depend on E, L and K. Specification of the cut-off L, coupling β, boundary condition h (and the already determined angular dependence) determine a specific set of solutions to equation (1).

Two independent solutions to equations (5) are given by $S_{\pm\gamma}$ with

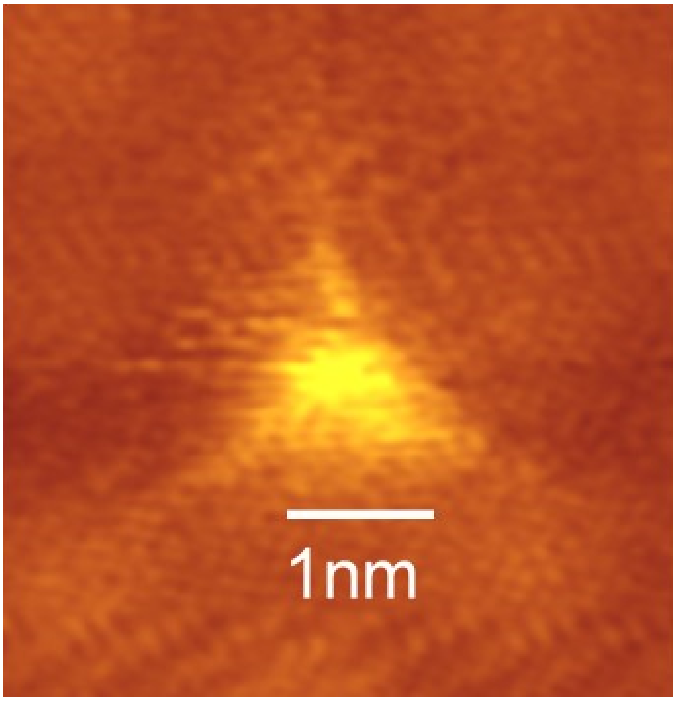

Supplementary Figure 3: **Characteristic topography signature of an isolated vacancy in graphene.** The triangular interference pattern arises due to the local crystal distortion and corresponding electronic state reconstruction. This is the feature that was used to identify single atom vacancies in this work.

$$\begin{aligned} S_\gamma(\rho) \equiv r^{\frac{1-d}{2}} e^{-\rho/2}\rho^{i\gamma} \Bigg[& {}_1F_1\left(i(\gamma+\beta);1+2i\gamma;\rho\right)\begin{pmatrix}1\\ i\end{pmatrix} \\ & + \frac{\gamma+\beta}{K}\,{}_1F_1\left(1+i(\gamma+\beta);1+2i\gamma;\rho\right)\begin{pmatrix}i\\ 1\end{pmatrix}\Bigg] \end{aligned} \tag{8}$$

where ${}_1F_1\,(a,b,z)$ is Kummer's function [3], $\gamma \equiv \sqrt{\beta^2-K^2}$ and $\rho \equiv 2iEr$. The $|E|\,r \ll 1$ behaviour of $S_{\pm\gamma}$ is given by

$$S_\gamma = r^{\frac{1-d}{2}}\rho^{i\gamma}\left(\begin{pmatrix}1+i\frac{\gamma+\beta}{K}\\ i+\frac{\gamma+\beta}{K}\end{pmatrix}+\mathcal{O}(|E|\,r)\right). \tag{9}$$

Solutions corresponding to outgoing and ingoing radial waves for $r\to\infty$ are given by the combinations

$$\begin{aligned} \psi_{\text{in}} &= \left(C_{\text{in}}^{\gamma}S_\gamma + C_{\text{in}}^{-\gamma}S_{-\gamma}\right) \\ &= r^{\frac{1-d}{2}}\left(\begin{pmatrix}1\\ -i\end{pmatrix}e^{-iEr-i\beta\log(2|E|r)}+\mathcal{O}\left(\frac{1}{|E|r}\right)\right) \\ \psi_{\text{sc}} &= \left(C_{\text{sc}}^{\gamma}S_\gamma + C_{\text{sc}}^{-\gamma}S_{-\gamma}\right) \\ &= r^{\frac{1-d}{2}}\left(\begin{pmatrix}1\\ i\end{pmatrix}e^{+iEr+i\beta\log(2|E|r)}+\mathcal{O}\left(\frac{1}{|E|r}\right)\right) \end{aligned} \tag{10}$$

where

$$\begin{aligned} C_{\text{sc}}^{\gamma} &\equiv Ke^{-\frac{\pi\beta}{2}}\frac{\left(e^{2\pi\beta}-e^{-2\pi\gamma}\right)\Gamma(i(\gamma+\beta))}{\left(e^{2\pi\gamma}-e^{-2\pi\gamma}\right)\Gamma(1+2i\gamma)} \\ C_{\text{in}}^{\gamma} &\equiv e^{\pi\gamma}e^{\frac{3\pi\beta}{2}}\frac{\left(e^{-2\pi\beta}-e^{-2\pi\gamma}\right)\Gamma(1+i\left(\gamma-\beta\right))}{\left(e^{2\pi\gamma}-e^{-2\pi\gamma}\right)\Gamma(1+2i\gamma)} \end{aligned} \tag{11}$$

For the case $E<0$. The log dependence in (10) is characteristic of the long range Coulomb tail and is irrelevant to the physics of the scattering problem [4]. Thus, a general solution to (5) can be written as

$$\begin{pmatrix}\Psi_1\\ \Psi_2\end{pmatrix} \propto \psi_{\text{in}}(\rho) + e^{2i\eta}\psi_{\text{sc}}(\rho) \tag{12}$$

where the scattering phase shift η is determined by the boundary condition (7) at $r=L$

$$e^{2i\eta(EL,h)} = -\left.\frac{\psi_{in,2}(\rho)-h\psi_{in,1}(\rho)}{\psi_{sc,2}(\rho)-h\psi_{sc,1}(\rho)}\right|_{\rho=2iEL} \tag{13}$$

The energy derivative of η is given by the logarithmic derivative of the RHS of (13)

$$\frac{d\eta}{dE} = \frac{1}{2i}\frac{d}{dE}\ln\left(-\frac{\psi_{in,2}(\rho) - h\psi_{in,1}(\rho)}{\psi_{sc,2}(\rho) - h\psi_{sc,1}(\rho)}\bigg|_{\rho=2iEL}\right) \tag{14}$$

The explicit expression is complicated and is therefore omitted. Resonant quasi-bound states appear for specific values of the energy E at which $d\eta/dE$ exhibits a sharp maxima. We would like to obtain an analytic expression for the position of the resonances in the regime $|E|\,L \ll 1$ appearing in Fig. 3 of the main text. Such an expression can in principle be extracted from (14). However, an easier route is available by allowing the energy parameter to be complex valued such that $E \to \varepsilon \equiv E_R - i\frac{W}{2}$ [5] and look for solutions of (5) and (7) with no e^{-iEr} plane wave solution for $r \to \infty$. The lifetime of the resonance is given by W^{-1} and is required to be positive. A solution with no ingoing wave is obtained by the requirement that $e^{-2i\eta(EL,h)} = 0$ or alternatively the vanishing of the denominator in (13) such that

$$h = \frac{\psi_{sc,2}(\rho)}{\psi_{sc,1}(\rho)}\bigg|_{\rho=2i\varepsilon L}. \tag{15}$$

Focusing our attention at the regime $|\varepsilon|\,L \ll 1$, one finds very different results depending on the value of β. For $\beta < \beta_c \equiv |K|$, γ is pure imaginary. Thus, from (9) and (10) we get that both $\psi_{sc,1,2} \propto \rho^{-\sqrt{K^2-\beta^2}}$ and therefore equation (15) is independent of ε to leading order in $|\varepsilon|\,L$. This inconsistency means that for fixed L and β there are no quasi bound states arbitrarily close to zero energy. In contrary, for $\beta > \beta_c$ and to leading order in $|\varepsilon|\,L$, equation (15) is given by

$$h_0 = \frac{C_s^{\gamma}\rho^{i\gamma}\left(i + \frac{\beta+\gamma}{K}\right) + (\gamma \to -\gamma)}{C_s^{\gamma}\rho^{i\gamma}\left(1 + i\frac{\beta+\gamma}{K}\right) + (\gamma \to -\gamma)} \tag{16}$$

where $h_0 \equiv h|_{E\to 0}$. Solving supplementary equation (16) for $\rho^{2i\gamma}$ gives $\rho^{2i\gamma} = z_0$ where $z_0 \equiv \frac{C_s^{-\gamma}((1-ih_0)(\beta-\gamma)-(h_0-i)K)}{C_s^{\gamma}((h_0-i)K-(1-ih_0)(\beta+\gamma))}$. Inserting $\rho \equiv 2i\varepsilon L$ and solving for ε yields

$$\varepsilon_n = -\epsilon_0 e^{i\Theta} e^{-\frac{\pi n}{\gamma}} \quad n \in \mathbb{Z} \tag{17}$$

where $\varepsilon_0 \equiv \frac{1}{2L}|z_0^{\frac{1}{2i\gamma}}| > 0$ and $\Theta \equiv \arg\left(iz_0^{\frac{1}{2i\gamma}}\right)$. The regime of validity of this result is for $n > n_{min}$, where n_{min} is such that $|\varepsilon_{n_{min}}|\,L \sim 1$. Using $\Gamma -$ function identities, the phase Θ of $-\varepsilon_n$ can be simplified to

$$\begin{aligned}\Theta &= \arg\left(iz_0^{\frac{1}{2i\gamma}}\right)\\ &= \frac{\pi}{2} - \frac{1}{2\gamma}\log(|z_0|)\\ &= -\frac{\pi}{2} + \frac{1}{4\gamma}\log\left(\frac{\sinh(\pi(\beta+\gamma))}{\sinh(\pi(\beta-\gamma))}\right).\end{aligned} \tag{18}$$

Note that Θ is independent on the boundary condition h_0. Recall that $\gamma \equiv \sqrt{\beta^2 - K^2}$, where K is given in (6) for $d \geq 2$. For $\beta > |K| \geq \frac{1}{2}$, $\arg(-\varepsilon_n)$ is a very slowly varying function bounded in the region $0 < \arg(-\varepsilon_n) < 0.046\pi$, as can be seen in supplementary figure 4. This is consistent with negative resonances $E_n = \mathrm{Re}(\varepsilon_n) < 0$ with width and $W_n \equiv -2\,\mathrm{Im}(\varepsilon_n) > 0$. Therefore, in the regime $|\varepsilon|\,L \ll 1$, ε_n describes a geometrically spaced set of infinitely many negative quasi bound states for all $\beta > |K|$, h, $d \geq 2$. The width of the resonance is given by $W_n \equiv -2\,\mathrm{Im}(\varepsilon_n) < 0.9\pi\,|\varepsilon_n|$ which describes resonances getting sharper indefinitely as $n \to \infty$. In the vicinity of the transition point $\beta - |K| \gtrsim 0$, $\arg(-\varepsilon_n) \cong -\frac{\pi}{1-e^{2|K|\pi}} + \mathcal{O}(\xi - |K|)^{1/2}$ which for $d = 2$, $m = 0 (\Leftrightarrow |K| = \frac{1}{2})$ agrees with the result of reference [6] in the main text.

Supplementary Note 2: Dirac Coulomb system as a model for a charged vacancy in graphene

As explained in the main text, we model excitations around the vacancy as massless Dirac particles and neglect any interactions between them. Therefore, for $r > L$, L being the characteristic size of the vacancy, they are described by the Dirac equation (5) with $d = 2$. The full spinor in this case is given in polar coordinates by

$$\psi(r,\phi) = e^{im\phi}\begin{pmatrix}\Psi_1(r)\\ i\Psi_2(r)e^{i\phi}\end{pmatrix} \tag{19}$$

in a representation where $H = \vec{\sigma}\cdot\vec{p} - \beta/r$. The components of the spinor correspond to amplitudes of the wave function on each of the two graphene sublattices. In the near vacancy region, $r < L$ we assume some unknown interaction that can be taken into account by the boundary condition (7) at $r = L$. Conventionally used choices are:

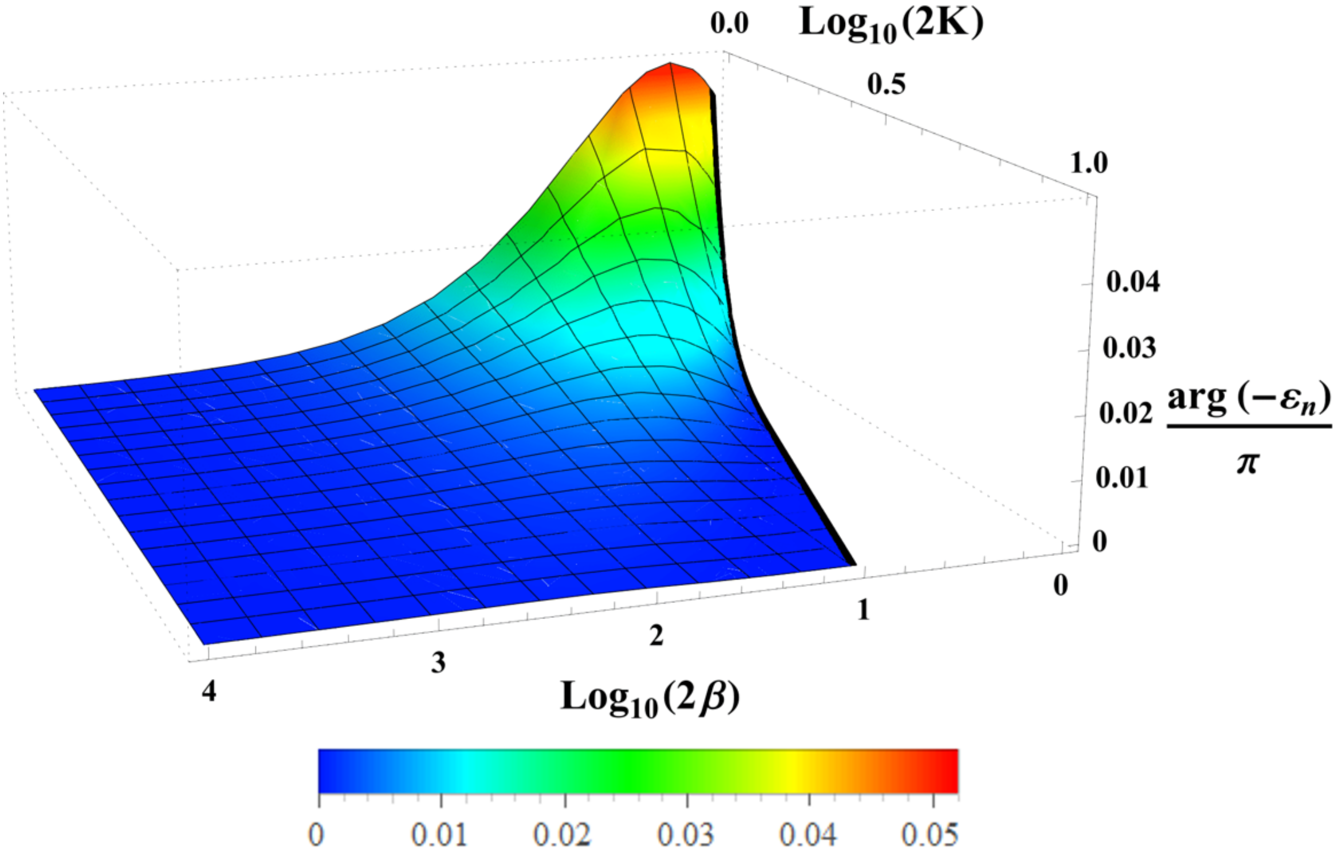

Supplementary Figure 4: **The phase of $-\varepsilon_n$ as a function of the coupling β and $|K|$ for $\beta > |K|$.** The bold black line represents $\xi = |K|$. It can be seen that the phase of $-\varepsilon_n$ takes values in the range $[0, 0.046\pi]$ for $d \geq 2$
.

(i) continuously connected constant potential $V_< = -\beta/L$ [7] corresponding to $h = J_{m+1}(\beta + EL)/J_m(\beta + EL)$, where $J_n(x)$ is Bessel's function; (ii) zero wavefunction on one of the graphene lattice sites [8] corresponding to $h = 0$; (iii) infinite mass term on boundary term [9] corresponding to $h = 1$. Generically, h can depend on E, L and m. From (6), the critical coupling is $\beta_c \equiv |m + 1/2| \leq 1/2$, giving rise to two angular momentum s-wave channels, $m = 0, -1$ for which $\beta_c = 1/2$.

An additional important property of our model is related to parity symmetry. Since mass or scalar potential terms are absent (and the Coulomb potential is radial), the Dirac equation in $2+1$ dimension is symmetric under 2 dimensional parity, P_y, in which $(x, y) \to (-x, y)$ [10]. The action of the parity operators on $\Psi(r, \phi)$ is defined as

$$\begin{aligned} \psi'(r,\phi) &\equiv P_y \psi(r, \pi - \phi) \\ &= \sigma_y \psi(r, \pi - \phi) \\ &= -i e^{i(-m-1)\phi} \begin{pmatrix} \Psi_2(r) \\ -i\Psi_1(r) e^{i\phi} \end{pmatrix}. \end{aligned} \tag{20}$$

This transformation can also be accounted for (up to an unimportant overall phase) by

$$\Psi_1(r) \to \Psi_2(r), \Psi_2(r) \to -\Psi_1(r), m \to -m - 1 \tag{21}$$

where $K \equiv m + 1/2$, thus $m \to -m - 1 \Leftrightarrow K \to -K$. Indeed, the Dirac equation (5), is invariant under (21).

From (21) it is apparent that solutions corresponding to angular momentum m and $-m-1$ are linked via parity symmetry. Since energy remains unchanged under parity, a natural question is whether the quasi bound energies are the same for angular momentum channels m and $-m-1$. The answer depends on the short distance region. For example, if we describe the $r < L$ regime by condition (i) then the quasi bound states will necessarily be degenerate over the m and $-m-1$ channels. The reason is that in this case, the potential of both regimes $r > L$, $r < L$ respects parity symmetry. As a result, h in (i) transforms like $\Psi_2(r)/\Psi_1(r)$ under (21), i.e., $h \to -h^{-1}$. Thus, by applying (21) on both side of (14) it is straightforward to obtain that $d\eta_m/dE = d\eta_{-m-1}/dE$. However, if the potential in the $r < L$ regime break parity, that is, the corresponding boundary condition does not transform as $h \to -h^{-1}$ under (21) (for example $h = 0, 1$), then $d\eta_m/dE \neq d\eta_{-m-1}/dE$ and the degeneracy will be broken. Specifically, for $m = 0, -1$ we find two interleaved geometric ladders of over critical states in the corresponding regime $\beta > \beta_c = 1/2$ as shown in the main text. The relative position of the two ladders typically depends on h and therefore is sensitive to the detail of the short range physics.

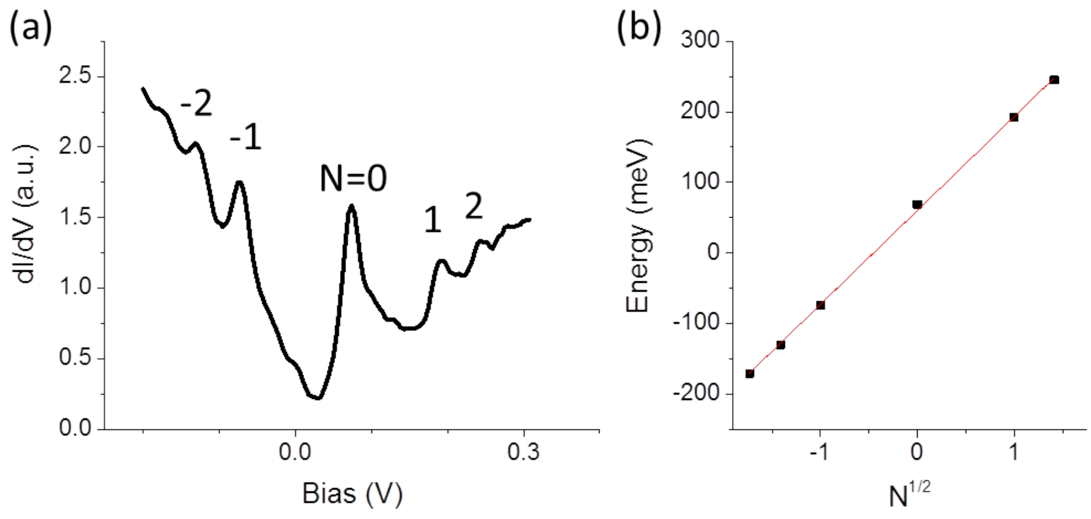

Supplementary Figure 5: **The Landau levels spectrum for twisted bilayer graphene under** 10T. (a) dI/dV curve on graphene at $B = 10$T with $V_b = -300$mV and $I = 20$pA. (b) Fit of the Landau level sequence in (a) used to extract the Fermi velocity.

All the figures in the main text describing the behaviour of $d\eta/dE$ as a function of E and the values of the quasi bound states $E_n(\beta)$ are extracted from the exact relation appearing in equation (14).

Supplementary Methods

Sample fabrication. In this work, we use G/G/BN on SiO_2 to perform the experiment. The hBN thin flakes were exfoliated onto the SiO_2 surface followed by a dry transfer process using a sacrificial PMMA thin film to stack the first graphene layer on the hBN flake. Before stacking the top layer graphene, the PMMA was removed with acetone and IPA, followed by furnace annealing in forming gas (10% H_2 and 90% Ar) at 230°C for 3 hours. The second layer graphene was stacked by using the same procedure as the first layer. Au/Ti electrodes were deposited by the standard SEM lithography for the STM contact. After the liftoff process, the sample was annealed again in furnace with forming gas to remove the PMMA residues. Subsequently, the sample is loaded in the UHV chamber for further annealing at $230°C$ overnight. To generate the single vacancies, the sample is exposed to a $100 - 140$eV He^+ ion beam followed by high temperature *in situ* annealing. The other stacked samples, G/BN/SiO_2 and G/G/SiO_2, were prepared by the same procedure.

Characterization by STM topography and Landau level (LL) spectroscopy. The STM experiment is performed at 4.2K using a cut PtIr tip. The dI/dV spectroscopy is performed using the standard lockin method with bias modulation typically 2mV at 473.1Hz. To charge the single vacancies, voltage pulses are applied directly at the desired vacancy site with the STM tip at ground potential. The intrinsic electronic properties of graphene can be effectively isolated from the random potential induced by the SiO_2 substrate by using an intermediate graphene layer and an hBN buffer underneath the layers. When the twist angle between the two stacked graphene layers exceeds 10° the two layers are electronically decoupled at the experimentally relevant energies. Therefore a large twist angle was chosen to ensure a linear dispersion near the Dirac point. LL spectroscopy provides a direct way to prove the layer decoupling. For single layer graphene, the energy level sequence is given by: $E_N = \mathrm{sign}(N) v_F \sqrt{2e\hbar |N| B}$, where N is the LL index, v_F is the Fermi velocity, $\hbar$ is the reduced Plank constant. For fixed magnetic field, the root N dependence for the sequence is the fingerprint of single layer graphene. supplementary figure 5(a) shows the LL spectrum of the G/G/BN sample at 10T. By fitting the LLs sequence (supplementary figure 5(b)), we obtain the value of the Fermi velocity $v_F = (1.2 \pm 0.02) \times 10^6 m/s$.

Supplementary References

[1] Dong, S.-H. *Wave Equations in Higher Dimensions* (Springer, 2011).

[2] Yang, C. N. Generalization of Sturm-Liouville theory to a system of ordinary differential equations with Dirac type spectrum. *Comm. Math. Phys.* **112**, 205–216 (1987).

[3] Abramowitz, M. & Stegun, I. A. *Handbook of mathematical functions: with formulas, graphs, and mathematical tables.* 55 (Courier Corporation, 1964).

[4] Walter Greiner, J. R., B. Mller. *Quantum Electrodynamics of Strong Fields* (Springer-Verlag Berlin Heidelberg, 1985).

[5] Friedrich, H. *Scattering Theory* (Springer-Verlag Berlin Heidelberg, 2013).

[6] Shytov, A. V., Katsnelson, M. I. & Levitov, L. S. Atomic Collapse and Quasi-Rydberg States in Graphene. *Phys. Rev. Lett.* **99**, 246802 (2007).

[7] Pereira, V. M., Kotov, V. N. & Castro Neto, A. H. Supercritical Coulomb impurities in gapped graphene. *Phys. Rev. B* **78**, 085101 (2008).

[8] Shytov, A. V., Katsnelson, M. I. & Levitov, L. S. Vacuum Polarization and Screening of Supercritical Impurities in Graphene. *Phys. Rev. Lett.* **99**, 236801 (2007).

[9] Pereira, V. M., Nilsson, J. & Castro Neto, A. H. Coulomb Impurity Problem in Graphene. *Phys. Rev. Lett.* **99**, 166802 (2007).

[10] WINKLER, R. & ZLICKE, U. Discrete symmetries of low-dimensional dirac models: A selective review with a focus on condensed-matter realizations. *The ANZIAM Journal* **57**, 3–17 (2015).
